# Predicting Heterosis and Selecting Superior Families and Individuals in *Fraxinus* spp. Based on Growth Traits and Genetic Distance Coupling

**DOI:** 10.3390/plants14162601

**Published:** 2025-08-21

**Authors:** Liping Yan, Chengcheng Gao, Chenggong Liu, Yinhua Wang, Ning Liu, Xueli Zhang, Fenfen Liu

**Affiliations:** 1 Shandong Provincial Academy of Forestry, Jinan 250014, China; ylp_982@163.com; 2 State Key Laboratory of Tree Genetics and Breeding, Research Institute of Forestry, Chinese Academy of Forestry, Beijing 100091, China; gaocc@caf.ac.cn (C.G.); ningpoplar@caf.ac.cn (N.L.); zxl961109@163.com (X.Z.); fen@caf.ac.cn (F.L.); 3 UGent-Woodlab (Laboratory of Wood Technology), Department of Environment, Ghent University, 9000 Ghent, Belgium

**Keywords:** *Fraxinus* spp., tree breeding, hybrids, genetic diversity, combining ability, molecular markers

## Abstract

*Fraxinus* spp. is one of the most important salt-alkali resistant tree species in the Yellow River region of China. However, the limited number of superior families and individuals, as well as the lack of a well-established parent selection system for hybrid breeding, have seriously constrained the improvement of seed orchards and the construction of advanced breeding populations. To address these issues, this study investigated 22 full-sib families of *Fraxinus* spp., using SSR molecular markers to calculate the genetic distance (*GD*) between parents. Combined with combining ability analysis, the study aimed to predict heterosis in offspring growth traits and select superior families and individuals through multi-trait comprehensive evaluation. The results showed the following: (1) Tree height (*TH*), diameter at breast height (*DBH*), and volume index (*VI*) exhibited extremely significant differences among families, indicating rich variation and strong selection potential. (2) The phenotypic and genotypic coefficients of variation for *TH*, *DBH*, and *VI* ranged from 4.34% to 16.04% and 5.10% to 17.73%, respectively. Family heritability was relatively high, ranging from 0.724 to 0.818, suggesting that growth is under strong genetic control. (3) The observed and expected heterozygosity of 15 parents were 0.557 and 0.410, respectively, indicating a moderate level of heterozygosity. Nei’s genetic diversity index and Shannon’s information index were 0.488 and 0.670, respectively, indicating relatively high genetic diversity. *GD* between parents ranged from 0.155 to 0.723. (4) Correlation analysis revealed significant or highly significant positive correlations between family heterosis and growth traits, combining ability, and *GD*, with specific combining ability (*SCA*) showing the strongest predictive power. Regression analysis further demonstrated significant linear correlations between *GD* and heterosis of *TH* and *VI*, and between *SCA* and heterosis of *TH*, *DBH*, and *VI*, establishing a *GD* threshold (≤0.723) and *SCA*-based co-selection strategy. In addition, four superior *Fraxinus* families and 11 elite individuals were selected. Their genetic gains for *TH*, *DBH*, and *VI* reached 2.28%, 3.30%, and 9.96% (family selection), and 1.98%, 2.11%, and 4.00% (individual selection), respectively. By integrating genetic distance (*GD*) and quantitative genetic combining ability (*SCA*), this study established a quantifiable prediction model and proposed the “*GD*–*SCA* dual-index parent selection method”, offering a new paradigm for genetic improvement in tree breeding.

## 1. Introduction

Ash trees (*Fraxinus* spp.) are broad-leaved deciduous trees in the genus *Fraxinus* (Oleaceae, Lamiales), exhibiting self- or cross-pollination and being either monoecious or dioecious [1]. They have a somatic chromosome number of 2n = 22 and belong to the “1A” cytological type [2]. *Fraxinus* species are widely distributed across Asia, the Americas, and Europe [3,4], demonstrating broad ecological adaptability. In China, they are important tree species for windbreaks, sand fixation, and the reclamation of saline-alkaline lands in the northern regions [5]. Among the most widely distributed species of *F. pennsylvanica*, *F. velutina* and *F. americana* exhibit high phenotypic plasticity, cold tolerance, salt tolerance, waterlogging tolerance, ease of vegetative propagation, and rapid growth [6,7,8]. They were introduced into northern and northeastern China in the mid-20th century as the parents of the breeding program for soil amelioration and ecological restoration [9,10]. In addition, due to their excellent wood properties and texture, they are widely used in the production of furniture, sporting goods, and tool handles [11]. However, owing to overharvesting, slow growth, and abiotic stresses such as drought and salinity, many *Fraxinus* species have been classified as Class II endangered plants in China [12,13]. With the increasing urgency of global sustainable forestry and the demand for high-quality tree breeding, the genetic improvement and conservation of *Fraxinus* germplasm resources face significant challenges [14,15].

Heterosis (*H*) is a widespread biological phenomenon and a fundamental basis of hybrid breeding [16,17]. Artificial hybrids derived from genetically diverse parents often exhibit traits superior to those of their parents, such as yield, growth rate, and stress resistance [18,19]. More importantly, distant hybridization among different species within the same genus can result in the integration of superior traits and the creation of novel germplasm [20]. Studies have shown that interspecific hybridization is an effective approach for developing fast-growing and high-quality *Fraxinus* cultivars [5]. For instance, clonal hybrids such as *F. mandshurica* × *F. americana* and *F. mandshurica* × *F. velutina* exhibit significantly higher growth rates and stress resistance than their maternal parents [21,22,23]. However, hybrid breeding practices indicate that trait improvement in plants is often goal-directed, and not all natural materials are suitable as ideal parents. Therefore, parent selection should follow high standards from the outset [24,25]. Strong combining ability is essential for effective parental selection and the development of superior hybrid combinations [26,27]. General combining ability (*GCA*) refers to the average performance of a parent in hybrid progeny, representing the additive genetic effects that can be stably inherited across generations [28]; specific combining ability (*SCA*), derived from allele interactions, reflects non-additive genetic effects that are heritable but not fixable, and is particularly important for the development of commercial hybrids [29,30,31]. Hence, parent selection based on both *GCA* and *SCA* is crucial for ensuring the stability and consistency of hybrid progeny, and for minimizing segregation and variability in subsequent generations [27,32].

In addition, genetic distance (*GD*) quantifies the genetic divergence between parents and serves as an important indicator in parent selection. With the advancement of modern biotechnology, predicting heterosis using *GD* calculated from molecular markers has become increasingly scientific and reliable [33,34]. Typically, there is a certain degree of correlation between *GD* and *H* in plant populations [35,36]. The improvement potential of hybrid progeny largely depends on the magnitude of *H* and the variability of *H* deviation. In general, the greater the genetic divergence between parental populations, the lower the variability in heterosis and the higher the overall heterotic effect [37]. In *Fraxinus* breeding, enhancing growth-related traits has become a key objective. However, the traditional approach—constructing numerous hybrid combinations and evaluating phenotypes—is labor-intensive, time-consuming, and costly. More notably, the lengthy process from hybridization to new variety development/quantitative maturity, taking years or decades, limits forest tree production [38]. Addressing this requires early identification of high-potential individuals for target traits, optimizing resources, reducing ineffective screening, and shortening the breeding cycle [39]. Previous studies have demonstrated that utilizing molecular markers to assess genetic distance, combined with combining ability for heterosis prediction, enhances early selection efficiency and accelerates breeding of adaptable varieties [40]. However, systematic reports on this technology for *Fraxinus* breeding remain limited.

To make up for this shortcoming, this study selected 15 *Fraxinus* parents with diverse genetic backgrounds and conducted an incomplete factorial mating design. Growth traits of 22 full-sib families were evaluated, genetic parameters were calculated, and SSR markers were used to estimate *GD* among the parents. The main objectives of this study were the following: (1) to evaluate the growth traits of 22 full-sib *Fraxinus* families, quantify the genetic variation among families, and assess their potential for genetic improvement; (2) to explore the relationships among *H*, *GCA*, *SCA*, and *GD*, in order to identify superior parents and predict progeny performance, thereby laying a theoretical foundation for accelerating *Fraxinus* improvement; and (3) to identify superior families and elite individuals based on multi-trait evaluation, providing materials for elite variety breeding and advanced-generation breeding population development. The results of this study will improve the efficiency and accuracy of elite parent selection in *Fraxinus* and offer valuable insights for the genetic improvement of other forest tree species.

## 2. Results

### 2.1. Genetic Variation in Growth Traits Among Full-Sib Fraxinus Families

The linear model effects and genetic parameters for tree height (*TH*), diameter at breast height (*DBH*), and volume index (*VI*) among full-sib *Fraxinus* families are presented in Table 1. The family variance components (*σ_F_*^2^) for *TH*, *DBH*, and *VI* ranged from 0.00279% to 21.70%, while the block × family interaction variances (*σ_BF_*^2^) ranged from 0.00022% to 3.71%. The environmental variance components (*σ_E_*^2^) were considerably high, particularly for *TH* (81.36%) and *DBH* (320.87%), indicating that environmental effects had a substantial influence on these traits. The phenotypic coefficient of variation (*PCV*) and genotypic coefficient of variation (*GCV*) ranged from 4.34% to 16.04% and from 5.10% to 17.73%, respectively. Among the traits, *VI* exhibited the highest level of genetic variation and family heritability, followed by *DBH* and *TH*. All three growth traits showed highly significant differences among full-sib families (*p* < 0.01), suggesting abundant genetic variation within the population.

**Figure 1:**
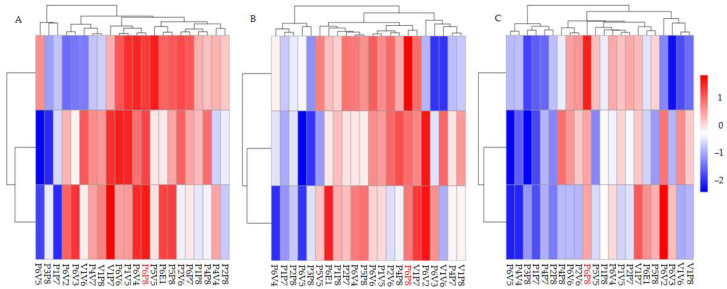
Heatmap of growth trait differences among families. Heatmaps about tree height (**A**), diameter at breast height (**B**) and volume index (**C**).

Table 2 and Figure 1 illustrate the growth performance and variation across families. The average *TH* across all families was 5.87 m, ranging from 5.25 m to 6.35 m. The average *DBH* was 7.04 cm, ranging from 5.86 cm to 8.00 cm. The average *VI* was 0.03293 m^3^, with a range of 0.02191 m^3^ to 0.04419 m^3^. Among all families, P6P8 exhibited the best growth performance, with phenotypic values of 6.35 m for *TH*, 7.99 cm for *DBH*, and 0.0412 m^3^ for *VI*, making it a highly promising candidate for selection as a superior family.

### 2.2. Analysis of Genetic Correlation Among Fraxinus Parents

As shown in Table 3, the number of alleles (*N_a_*) amplified by each pair of SSR primers ranged from 1.667 to 3.333, with a total of 34 alleles amplified. On average, each primer pair amplified 2.429 alleles, and the average effective number of alleles (*N_e_*) was 1.977. Among them, seven primer pairs had a polymorphic information content (*PIC*) greater than 0.5, indicating a relatively high level of polymorphism. The observed heterozygosity (*H_o_*) ranged from 0.167 to 1.000, with an average of 0.557, while the expected heterozygosity (*H_e_*) ranged from 0.178 to 0.585, with an average of 0.410, reflecting a moderate level of heterozygosity among the parental genotypes. Nei’s genetic diversity index (*h*) ranged from 0.242 to 0.718 (average 0.488), and Shannon’s information index (*I*) ranged from 0.326 to 0.981 (average 0.670), indicating a high level of genetic diversity among the tested parents.

As shown in Table 4, the genetic distance (*GD*) between the parents of the 22 families ranged from 0.155 to 0.723, with an average *GD* of 0.286. Some hybrid combinations shared similar *GD* values. The greatest genetic distance (0.723) was observed between the parental combinations of P6E1 and P4P7. In contrast, nine families with V5 or P8 as male parents exhibited the lowest and most similar *GD* values (0.155), suggesting that these parental combinations were more closely related.

### 2.3. Analysis of Heterosis and Combining Ability for Growth Traits Among Fraxinus Families

The degree of heterosis varied considerably among families for all traits (Table 5), with the greatest variation observed for the volume index (*VI*), ranging from –40.11% to 73.67%. Among the progeny, the combinations P2V6, P2P7, and P5P8 exhibited the highest heterosis for tree height (*TH*), at 20.87%, 18.62%, and 25.53%, respectively. For diameter at breast height (*DBH*), the highest heterosis was observed in P5V5 (21.45%), P2V6 (15.87%), and P5P8 (21.46%). Similarly, for *VI*, P5V5 (62.15%), P2V6 (65.21%), and P5P8 (73.67%) showed the highest values. Notably, P2V6 and P5P8 exhibited strong heterosis across all three traits, indicating their potential as superior hybrid combinations.

The combining ability for *TH*, *DBH*, and *VI* is presented in Table 6. Among the maternal parents, combinations involving P6, P2, V1, and P5 had positive *GCA_P1_* values for *TH*, *DBH*, and *VI* in seven, two, three, and two combinations, respectively. Notably, only the P2P8 combination showed a positive *GCA_P1_* for *DBH*, while the *GCA_P1_* values for its other traits were negative. Among the five combinations with P6 as the female parent (P6V4, P6V5, P6V2, P6V6, and P6P8), *GCA_P1_* values for *TH* and *VI* were the highest, reaching 0.29 and 0.0035, respectively. For *DBH*, the highest *GCA_P1_* values were observed in P5V5 and P5P8 (both 0.26), indicating that P6 and P5 are among the most promising female parents for breeding purposes.

Six combinations had positive *GCA_P2_* values for *TH*, *DBH*, and *VI* simultaneously, with P7 and V6 as the male parents. Only two combinations (P6E1 and P4P7) showed positive *GCA_P2_* values for both *TH* and *DBH*. Combinations with P8 and V2 as male parents had positive *GCA_P2_* for *TH* and *VI*, while those with V4 and V5 had positive *GCA_P2_* values only for *DBH*. Among these, P6V2 showed the highest *GCA_P2_* values for *TH* (0.78) and *VI* (0.0091), and combinations involving V6 as the male parent (P6V6, P2V6, and V1V6) had the highest *GCA_P2_* values for *DBH* (0.15), suggesting that V2 and V6 are the most promising male breeding parents.

In terms of overall general combining ability (*GCA*), among the 22 families, P6V2 showed the highest *GCA* for *TH* (1.07) and *VI* (0.0127), while P6P8 had the highest *GCA* for *DBH* (0.97), indicating that these hybrids are capable of producing vigorous growth performance. Regarding specific combining ability (*SCA*), P6P8 exhibited the highest *SCA* values across all three traits—*TH* (0.97), *DBH* (0.37), and *VI* (0.0129)—highlighting its potential as a superior cross for the development of high-growth *Fraxinus* cultivars.

### 2.4. Correlation and Regression Analysis

**Figure 2:**
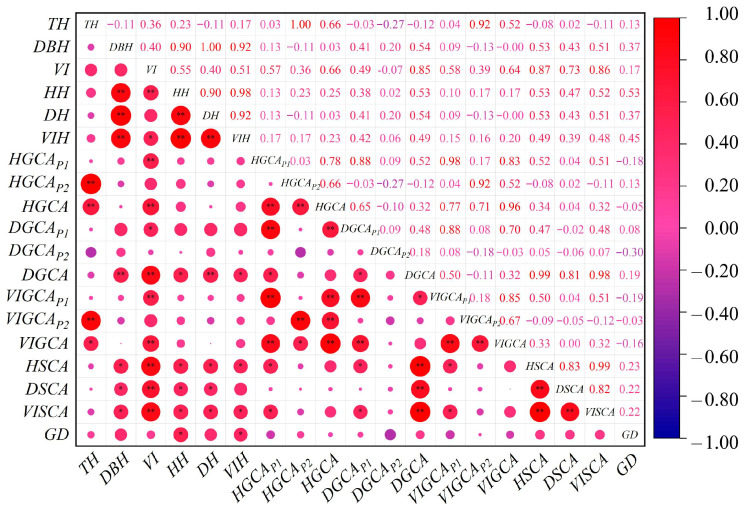
Correlation between combining ability, genetic distance, and heterosis degree of hybrid progeny. *HH* stands for tree height heterosis degree, *DH* stands for diameter at breast height heterosis degree, *VIH* stands for the volume index heterosis degree, *GCA_P1_* stands for parental general performance, *GCA_P2_* stands for parental strain’s general performance coefficient, *GCA* stands for general combining ability, *SCA* stands for specific combining ability and *GD* stands for genetic distance. * means the probability of significance is 0.05, and ** means the probability of significance is 0.01.

Correlation analysis (Figure 2) showed that genetic distance (*GD*) between parents was positively correlated with the heterosis degree of all traits, and the correlations with tree height (*TH*) heterosis and volume index (*VI*) heterosis were statistically significant (*p* < 0.05). Both general combining ability (*GCA*) and specific combining ability (*SCA*) were positively correlated with the heterosis degree of the corresponding traits. Specifically, the correlation between SCA and heterosis was significant for *TH* (*p* < 0.05), *DBH* (*p* < 0.05), and *VI* (*p* < 0.05). Only the correlation between *DBH*, *GCA*, and *DBH* heterosis was extremely significant (*p* < 0.01). These results suggest that, compared to *GCA*, heterosis performance in hybrid progeny is more strongly associated with *GD* and *SCA*.

**Figure 3:**
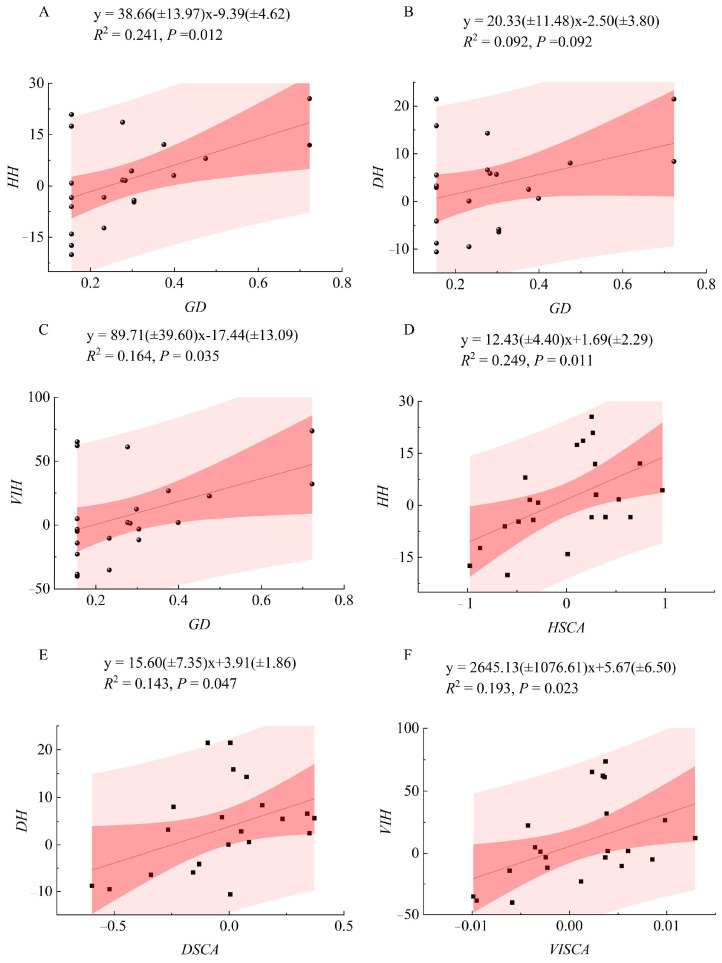
Linear regression analysis between specific combining ability, genetic distance, and heterosis degree of hybrid progeny. x represents the corresponding horizontal coordinate, y represents the corresponding vertical coordinate, *R*^2^ is the coefficient of determination, and *P* indicates significance. (**A**–**C**) represent the regression relationships between the *GD* among parents and the heterosis degree for *TH*, *DBH*, and *VI*, respectively. (**D**–**F**) represent the regression relationships between the specific combining ability of *TH*, *DBH*, and *VI* and their corresponding heterosis degree, respectively.

Regression analysis (Figure 3) revealed linear positive relationships between heterosis degrees of growth traits and both parental *GD* and *SCA*. Significance tests of the linear regression models indicated that, except for the non-significant relationship between *GD* and *DBH* heterosis, all other models—including *TH* and *VI* heterosis vs. *GD* and heterosis vs. *SCA* for all traits—were statistically significant (*p* < 0.05). Among them, the regression between *SCA* and *TH* heterosis showed the best fit, with the highest coefficient of determination (*R*^2^ = 0.249). Scatter plots showed that heterosis values increased with rising *GD* and *SCA*, followed by a decline, indicating a nonlinear trend.

Within the confidence interval, the highest heterosis was observed when *GD* (range: 0.155–0.723) approached approximately 0.7. For *SCA* of tree height (range: −0.98–0.97), the optimal range was 0.5–1.0; for *DBH* (range: −0.60–0.37), the optimal *SCA* range was 0.0–0.25; and for *VI* (range: 0.0099–0.0129), the highest heterosis occurred when *SCA* was between 0.005 and 0.010.

### 2.5. Selection of Superior Families and Elite Individuals in Fraxinus

A comprehensive evaluation of all traits was conducted across the 22 full-sib families. The *Q_i_* values for each family are shown in Table 7. The highest *Q_i_* value (1.732) was observed in family P6P8, while the lowest (1.438) was found in P6V5. Based on a 20% selection intensity threshold, four families were selected as superior: P6P8, V1P7, P5P8, and P6V6. The average tree height (*TH*), diameter at breast height (*DBH*), and volume index (*VI*) of the selected families were 6.25 m, 7.71 cm, and 0.04076 m^3^, respectively—representing increases of 6.34%, 9.63%, and 23.78% over the population means. The corresponding genetic gains were 2.28% (*TH*), 3.30% (*DBH*), and 9.96% (*VI*).

Using a more stringent 1% selection intensity threshold, 11 elite individual trees were selected from the entire population (Table 8). Compared to the overall means, the selected individuals exhibited average increases of 23.36% in *TH*, 66.35% in *DBH*, and 199.61% in *VI*. The corresponding genetic gains for these individuals were 1.98% (*TH*), 2.11% (*DBH*), and 4.00% (*VI*).

## 3. Discussion

Genetic variation is the prerequisite for genetic improvement in plants, and its effectiveness determines the potential of a species in long-term breeding programs [41]. Understanding the patterns of genetic variation is fundamental to selecting appropriate breeding strategies [42,43]. Moreover, tree age correlation indicates growth stability by measuring juvenile–mature trait consistency. When strong, it enables effective early selection for mature performance, improving genetic breeding efficiency [44,45]. *Fraxinus* data confirm this: genetic height (0.89) and diameter (0.92) correlations are high at age 7–8 (*F. excelsior*) [46], while juvenile–mature volume and progeny height adult volume correlations are significant (*F. mandshurica*) [47]. This demonstrates early growth stability and supports early selection feasibility. In this study, highly significant differences in growth traits were observed among 8-year-old full-sib families of *Fraxinus*, indicating strong genetic control over progeny traits [48]. Heritability analysis showed that family heritability values exceeded 0.7, indicating a high level of genetic control [31]. These values are much higher than the heritability of tree height (0.30) and diameter at breast height (0.27) observed in 8-year-old *F. excelsior* progeny [46]. Moreover, family-level heritability was higher than individual-tree heritability, indicating that greater genetic gains can be achieved through family selection in *Fraxinus*, consistent with the findings of Weng et al. [49]. These results suggest that hybridizing the selected *Fraxinus* parents can create a base population with sufficient variation for the next generation, providing opportunities for selecting superior families.

Heterosis is the phenomenon where hybrids resulting from crosses between genetically distinct parents outperform their parents in various traits [16]. It is one of the key innovations in modern plant breeding and genetic improvement, playing an important role in global wood and food security [50]. However, the superiority of hybrids depends not only on the performance of the parents but also on the combining ability between them [51]. Combining ability analysis is an effective method to evaluate and select superior parents and hybrid combinations, thereby producing large numbers of high-quality families or progeny [52,53]. In this study, maternal parent P6 and paternal parent V6 showed high general combining ability (*GCA*) values for growth traits. The hybrids from these parents, such as P6V6, P6P8, P6V4, and P2V6, exhibited high specific combining ability (*SCA*) values and significant heterosis, suggesting that strong heterotic combinations are more likely when at least one parent has high *GCA* or when the combination has high *SCA*—consistent with results in maize [54], Camellia oleifera [55], and *Populus tomentosa* [56]. Therefore, when breeding for superior *Fraxinus* genotypes, it is advisable to select parents with both high *GCA* and high *SCA* values to increase the probability of producing highly heterotic combinations.

Furthermore, this study found that in some highly heterotic combinations—such as P2P7 and P2V6 (with high heterosis in tree height), and P5P8 and P5V5 (with high heterosis in *DBH*)—the parental *GCA* values were high, while *SCA* values were not. Conversely, although the combination P6P8 showed the highest *GCA* and *SCA* values for all three growth traits, its heterosis performance was weak. This indicates that forming highly heterotic combinations in *Fraxinus* cannot rely solely on parental *GCA* or hybrid *SCA*; instead, a comprehensive evaluation of individual traits and their contributing factors is necessary. This is consistent with the findings of Josue et al. [57] in maize. Additionally, correlation analysis between heterosis and combining ability in this study revealed that only *DBH* heterosis was significantly positively correlated with *GCA*, while all three traits showed significant positive correlations with *SCA*, with higher correlation coefficients—indicating that *SCA* plays a more important predictive role for growth-related traits in *Fraxinus*. This is in line with findings by Umakanth et al. [58] in sweet sorghum, where *SCA* variance components for total biomass, juice yield, and grain yield were greater than *GCA*, and by Chai et al. [59] in *C. oleifera*, where *SCA* was more important than *GCA* for most traits. However, in practical breeding, parental selection should consider both *GCA* and *SCA* along with the genetic background of the parental material to increase the likelihood of producing superior hybrids.

In recent years, with the rapid development of molecular markers, breeders have commonly used molecular marker-based genetic distance to predict heterosis in crops. Since its first application in *Zea mays* L. [60], significant progress has been made in tree species such as *Populus* [61], *Salix* [36], *Pinus* [62], and *Liriodendron chinense* [63] for predicting heterosis in traits such as growth and resistance. Simple sequence repeat (SSR) markers or microsatellites have been considered markers of choice due to their co-dominant inheritance, high polymorphism, multi-allelic nature, and high reproducibility [64,65,66]. In this study, the average genetic distance (*GD*) among parents based on SSR markers was 0.286, which is considered moderate compared to previous studies [62,67]. Among strong heterotic combinations, P2P7 and P2V6 had relatively large parental *GD* values, whereas combinations like P5V5 and P5P8 had smaller *GD*s yet showed strong heterosis. Correlation analysis showed that *GD* was significantly positively correlated with heterosis in tree height and volume index, but not *DBH*, suggesting that greater parental *GD* tends to result in stronger heterosis. This finding aligns with studies in *Larix decidua* [68], *chili pepper* [69], and *Pennisetum glaucum* [70], but contrasts with studies in *Cucumis melo* [71], certain *Pennisetum glaucum* lines [72], and *Brassica napus* [73]. These discrepancies may be attributed to differences in plant materials, marker types and quantities, parental genetic relationships, phenotypic traits, and environmental factors. The observed positive relationship between *GD* and heterosis may be explained by either (1) increased distance resulting from the accumulation of favorable alleles, or (2) greater differences in gene frequencies at key loci between parental populations as *GD* increases [74,75]. Our results suggest that *GD* can be used to predict heterosis in tree height and volume index. However, the relationship between *GD* and heterosis is complex and requires further investigation. Moreover, due to the limited number of SSR markers used in this study, it is difficult to fully represent genome-wide information. Future work may incorporate higher-density markers (e.g., SNPs) or genome-wide association studies to improve the accuracy of heterosis prediction.

## 4. Materials and Methods

### 4.1. Test Materials

**Figure 4:**
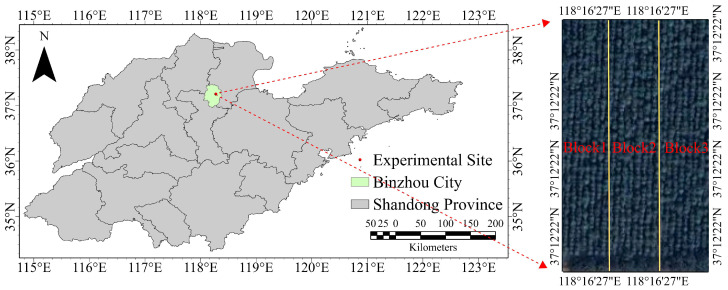
Location of the experimental site and overview of the experimental forest.

The experimental materials consisted of eight-year-old full-sib families of *Fraxinus* established in a trial plantation. In October 2015, seeds from 22 full-sib families were obtained through an incomplete factorial-controlled pollination design; details of the parental lines are shown in Table 9. It is important to emphasize that all the hybrid parents discussed in this study are superior varieties that have been selected by our team or collaborating units. This selection process followed preliminary evaluations of phenotypic traits, including tree height, diameter at breast height, and salt-alkali tolerance. In March 2016, the seeds were sown in non-woven container bags at the nursery of the Shandong Academy of Forestry. In March 2017, one-year-old seedlings (average basal diameter ~6.11 cm) were transplanted to the experimental base of Bohua Ecological Agriculture Co., Ltd., Shandong Province (Shandong, China). The site is located on the Yellow River alluvial plain, at an elevation of 9.66 m with a slope gradient of less than 1.75%. It has a temperate continental monsoon climate, with an annual average temperature of 13.6 °C, a frost-free period of 191 days, 2479.8 h of annual sunshine, average annual precipitation of 582.8 mm, and an annual evaporation of 1686.6 mm. The soil type is salinized fluvo-aquic soil with a pH of 8.48, salt content of 3.43 g·kg^−1^, available nitrogen 121.23 mg·kg^−1^, available phosphorus 32.02 mg·kg^−1^, and available potassium 149.67 mg·kg^−1^ [76]. Figure 4 shows the trial site location and general overview. A randomized complete block design was applied, with three blocks in total. Each plot contained 20 trees planted at a spacing of 3 m × 4 m.

### 4.2. Growth Trait Measurements

In November 2023 (after leaf fall in autumn), tree height (*TH*) and diameter at breast height (*DBH*) were measured for 22 eight-year-old full-sib families. Measurements were conducted using a tower ruler (accuracy: 0.01 m; Saiwei Geological Survey and Mapping Co., Ltd., Xuzhou, China) and a vernier caliper (accuracy: 0.01 cm; Mitutoyo Precision Measuring Instruments Co., Ltd., Shanghai, China). Survival rates for each family are shown in Table 10.

### 4.3. DNA Extraction and SSR Analysis

Young leaves from the current-year shoots of 15 parent trees were collected for DNA extraction and SSR marker analysis. Genomic DNA was extracted using a modified CTAB method, in which 2% β-mercaptoethanol and 2% PVP were added to the extraction buffer [77]. DNA concentration and purity were assessed using a NanoDrop-2000 ultramicro spectrophotometer (Thermo Fisher Scientific, Waltham, MA, USA), and the samples were diluted to 30 ng·μL^−1^ and stored at −20 °C for further use.

A total of 14 SSR primer pairs were selected from 42 pairs previously developed by the research group and synthesized by Shandong VON Biotechnology Co., Ltd. (Yantai, China) (Table S1). Fluorescently labeled PCR amplification and capillary electrophoresis were conducted following the procedure described by Yan [78]. Raw electropherogram files were imported into GeneMarker (Version 2.2.0, SoftGenetics LLC, State College, PA, USA) for fragment scoring and allele peak analysis.

### 4.4. Data Processing

A nested PCR product size was read from the amplified DNA samples. SSR genotype data were formatted using GeneAlEx (Version 6.503, Australian National University, Canberra, Australia) to calculate the number of alleles (*N_a_*), effective number of alleles (*N_e_*), observed heterozygosity (*Ho*), expected heterozygosity (*H_e_*), Nei’s genetic diversity index (*h*), and Shannon’s information index (*I*). Polymorphism information content (*PIC*) and genetic distance (*GD*) were computed using PowerMarker (Version 3.25, North Carolina State University, Raleigh, NC, USA).

Statistical analyses of phenotypic traits were conducted using SPSS (Version 21.0), where ANOVA F-tests were used to assess the significance of fixed effects. Trait data were organized in Excel (Version 2016) (Table 11). Origin (Version 2021b) was used to evaluate the phenotypic values and heterosis of hybrid progenies, calculate correlation coefficients between heterosis and parental combining ability or genetic distance, construct scatterplots illustrating the relationships between heterosis and parental parameters, assess the goodness-of-fit of simple linear regression models, and generate the corresponding regression equations.

## 5. Conclusions

In this study, we conducted a comprehensive analysis of growth traits in 22 full-sib families of *Fraxinus* spp., clarifying the patterns of genetic variation, combining ability effects, heterosis expression, and their relationship with parental genetic distance. The main conclusions are as follows: tree height (*TH*), diameter at breast height (*DBH*), and volume index (*VI*) showed highly significant differences among families, with family heritability values exceeding 0.7, indicating strong genetic control over growth traits and the potential for early selection. Heterosis was closely associated with combining ability, with specific combining ability (*SCA*) showing significantly positive correlations with heterosis across all three traits and providing better predictive value than general combining ability (*GCA*). SSR analysis revealed that genetic distance (*GD*) among parents was moderate, averaging 0.286. *GD* was significantly positively correlated with heterosis in *TH* and *VI*, demonstrating its reference value for heterosis prediction. Four elite families (P6P8, V1P7, P5P8, P6V6) and eleven superior individuals were selected, with growth traits exceeding the overall mean by 6.34%, 9.63%, and 23.78%, respectively, and genetic gains reaching 2.28%, 3.30%, and 9.96%. Outstanding parental lines included maternal parents P6, P2, P5, and paternal parents V6, V2, all exhibiting high *GCA* and *SCA* effects, making them suitable as core parents for advanced-generation breeding. The eight selected individuals exhibited improvements of 23.36%, 66.35%, and 199.61% in *TH*, *DBH*, and *VI*, respectively, with corresponding genetic gains of 1.98%, 2.11%, and 4.00%, demonstrating high breeding value. In conclusion, intra-specific hybridization in *Fraxinus* exhibits notable heterosis. Coordinated prediction of superior combinations using *GD* and *SCA* is a feasible strategy for enhancing breeding efficiency and provides technical support for elite parent selection and germplasm improvement.

## Figures and Tables

**Table 1:** Variance components and genetic parameters of different traits. *TH* tree height, *DBH* diameter at breast height, *VI* volume index, *σ_F_*^2^ variance components of family, *σ_BF_*^2^ variance components of interaction of block and family, *σ_E_*^2^ variance components of environment. ** *F* value  <  0.01. The block degrees of freedom and the family degrees of freedom are 3 and 21, respectively. The units of each trait—*TH* (m), *DBH* (cm), *VI* (m^3^), coefficient of variation (%)—are the same for tables below. *PCV* phenotypic coefficient of variation, *GCV* genotypic coefficient of variation, *FH* family heritability, *SH* single tree heritability.

Traits	*σ_F_*^2^ (%)	*σ_BF_*^2^ (%)	*σ_E_*^2^ (%)	*PCV* (%)	*GCV* (%)	*FH*	*SH*
*TH*	6.49 **	3.71	81.36	4.34	5.10	0.724	0.284
*DBH*	21.70 **	2.38	320.87	6.62	7.43	0.793	0.252
*VI*	0.00279 **	0.00022	0.03603	16.04	17.73	0.818	0.286

**Table 2:** The average performance of 22 hybrid combinations.

Family	*TH* (m)	*DBH* (cm)	*VI* (m^3^)	Family	*TH* (m)	*DBH* (cm)	*VI* (m^3^)
P1P7	5.29 ± 0.82	6.24 ± 1.26	0.02240 ± 0.01	P5V5	5.98 ± 0.83	6.96 ± 1.91	0.03249 ± 0.02
P1P8	5.78 ± 1.15	7.17 ± 1.97	0.03409 ± 0.02	P6E1	5.89 ± 0.94	7.12 ± 1.74	0.03325 ± 0.02
P1V5	6.26 ± 0.69	7.30 ± 1.52	0.03571 ± 0.02	P6P8	6.35 ± 0.70	8.00 ± 1.91	0.04419 ± 0.02
P2P7	5.96 ± 1.00	7.26 ± 1.92	0.03557 ± 0.02	P6V2	5.63 ± 1.08	7.53 ± 2.63	0.03888 ± 0.03
P2P8	5.62 ± 0.91	6.54 ± 1.73	0.02717 ± 0.02	P6V3	5.64 ± 1.30	6.47 ± 1.98	0.02833 ± 0.02
P2V6	6.03 ± 0.85	7.34 ± 1.84	0.03617 ± 0.02	P6V4	6.30 ± 0.86	7.38 ± 1.57	0.03715 ± 0.02
P3P8	5.25 ± 0.87	6.06 ± 1.57	0.02191 ± 0.01	P6V5	5.31 ± 1.16	5.86 ± 1.86	0.02206 ± 0.02
P4P7	5.79 ± 0.73	6.71 ± 1.10	0.02732 ± 0.01	P6V6	6.22 ± 0.96	7.47 ± 1.89	0.03859 ± 0.02
P4P8	5.84 ± 1.06	7.26 ± 1.89	0.03505 ± 0.02	V1P7	6.26 ± 0.85	7.81 ± 1.86	0.04178 ± 0.02
P4V4	5.85 ± 0.88	6.64 ± 1.46	0.02778 ± 0.01	V1P8	5.84 ± 0.81	6.91 ± 1.78	0.03122 ± 0.02
P5P8	6.16 ± 0.60	7.58 ± 2.15	0.03850 ± 0.03	V1V6	5.64 ± 1.31	6.43 ± 2.45	0.03000 ± 0.03
Average of 22 families					5.87 ± 0.97	7.04 ± 1.86	0.03293 ± 0.02

**Table 3:** Polymorphic analysis of the 14 SSR markers.

Marker	Number of Alleles (*N_a_*)	Effective Number of Alleles (*N_e_*)	Polymorphic Information Content (*PIC*)	Observed Heterozygosity (*H_o_*)	Expected Heterozygosity (*H_e_*)	Nei’s Gene Diversity Index (*h*)	Shannon’s Information Index (*I*)
SSR 82	2.000	1.276	0.325	0.214	0.178	0.357	0.326
SSR 93	2.667	2.118	0.535	0.548	0.410	0.580	0.716
SSR 95	2.333	2.051	0.507	0.381	0.433	0.549	0.683
SSR 112	2.000	1.518	0.294	0.437	0.317	0.331	0.485
SSR 120	2.667	2.190	0.512	0.667	0.407	0.592	0.704
SSR 144	2.333	2.222	0.421	1.000	0.542	0.531	0.809
SSR 147	2.000	1.756	0.260	0.333	0.333	0.287	0.534
SSR 167	1.667	1.649	0.342	0.167	0.329	0.380	0.457
SSR 186	2.667	2.200	0.549	0.833	0.514	0.611	0.833
SSR 187	2.667	2.200	0.549	0.833	0.514	0.611	0.833
SSR 202	3.333	2.493	0.670	0.833	0.553	0.718	0.962
SSR 203	3.333	2.517	0.646	0.944	0.585	0.696	0.981
SSR 208	2.000	1.616	0.232	0.222	0.259	0.242	0.442
SSR 213	2.333	1.871	0.330	0.389	0.370	0.347	0.621
Mean	2.429	1.977	0.441	0.557	0.410	0.488	0.670

**Table 4:** Parental genetic distance (*GD*).

Serial Number	Family	*GD*	Serial Number	Family	*GD*
1	P6V5	0.155	12	P1P7	0.277
2	P5V5	0.155	13	P2P7	0.277
3	P3P8	0.155	14	P6V3	0.283
4	P1V5	0.155	15	V1V6	0.298
5	P6P8	0.155	16	P6V6	0.304
6	P1P8	0.155	17	P2V6	0.304
7	P2P8	0.155	18	V1P8	0.375
8	P4P8	0.155	19	P6V2	0.399
9	P5P8	0.155	20	V1P7	0.475
10	P6V4	0.233	21	P6E1	0.723
11	P4V4	0.233	22	P4P7	0.723
Average of 22 families					0.286

**Table 5:** Heterosis of hybrid progeny growth traits. *HH*, *DH*, and *VIH*, respectively, represent the hybrid superiority degrees of tree height, diameter at breast height, and volume index.

Family	Heterosis Degree (*HH*) (%)	Heterosis Degree (*DH*) (%)	Heterosis Degree (*VIH*) (%)	Family	Heterosis Degree (*HH*) (%)	Heterosis Degree (*DH*) (%)	Heterosis Degree (*VIH*) (%)
P1P7	−12.32	−9.51	−35.21	P5V5	17.46	21.45	62.15
P1P8	3.04	0.62	1.96	P6E1	−3.38	0.08	−10.40
P1V5	1.71	6.60	1.99	P6P8	4.36	5.66	12.39
P2P7	18.62	14.3	61.13	P6V2	0.78	−4.09	4.87
P2P8	8.02	8.04	22.60	P6V3	−14.07	−4.17	−22.88
P2V6	20.87	15.87	65.21	P6V4	−3.40	5.52	−4.99
P3P8	−17.41	−10.61	−38.61	P6V5	−20.10	−8.77	−40.11
P4P7	1.60	5.87	1.30	P6V6	−3.44	2.88	−3.32
P4P8	11.91	8.37	32.12	V1P7	12.09	2.51	26.81
P4V4	−6.07	3.26	−14.20	V1P8	−4.20	−5.88	−11.66
P5P8	25.53	21.46	73.67	V1V6	−4.70	−6.41	−3.24
Average of 22 families					1.68	3.32	8.25

**Table 6:** Combining ability of hybrid combination progeny traits. *GCA_P1_*, *GCA_P2_*, *GCA*, and *SCA*, respectively, represent the general compatibility of the maternal parent, the general compatibility of the paternal parent, the general compatibility, and the special compatibility.

Family	*TH*				*DBH*				*VI*			
	*GCA_P1_*	*GCA_P2_*	*GCA*	*SCA*	*GCA_P1_*	*GCA_P2_*	*GCA*	*SCA*	*GCA_P1_*	*GCA_P2_*	*GCA*	*SCA*
P1P7	−0.01	0.17	0.16	−0.87	−0.04	0.01	−0.87	−0.52	−0.0012	0.0012	0.0000	−0.0099
P1P8	−0.01	0.10	0.09	0.30	−0.04	−0.01	0.29	0.09	−0.0012	0.0004	−0.0008	0.0039
P1V5	−0.01	−0.22	−0.24	0.53	−0.04	0.09	0.62	0.34	−0.0012	−0.0017	−0.0029	0.0060
P2P7	0.16	0.17	0.33	0.17	0.05	0.01	0.17	0.08	0.0010	0.0012	0.0022	0.0036
P2P8	−0.25	0.10	−0.15	−0.42	0.05	−0.01	−0.42	−0.24	−0.0039	0.0004	−0.0035	−0.0043
P2V6	0.16	0.21	0.37	0.27	0.05	0.15	0.41	0.02	0.0010	0.0034	0.0044	0.0023
P3P8	−0.89	0.10	−0.79	−0.98	−0.60	−0.01	−0.98	0.01	−0.0103	0.0004	−0.0099	−0.0095
P4P7	−0.25	0.08	−0.17	−0.37	−0.10	0.03	−0.34	−0.03	−0.0039	−0.0011	−0.005	−0.0030
P4P8	−0.25	0.10	−0.15	0.29	−0.10	−0.01	0.28	0.14	−0.0039	0.0004	−0.0035	0.0038
P4V4	−0.25	−0.18	−0.42	−0.62	−0.10	0.13	−0.50	−0.27	−0.0039	−0.0021	−0.006	−0.0062
P5P8	0.11	0.10	0.21	0.26	0.26	−0.01	0.25	0.01	0.0017	0.0004	0.0021	0.0037
P5V5	0.11	−0.22	−0.12	0.11	0.26	0.09	0.20	−0.09	0.0017	−0.0017	0.0001	0.0034
P6E1	0.29	0.08	0.37	0.39	0.13	0.03	0.43	0.00	0.0035	−0.0011	0.0024	0.0054
P6P8	0.29	0.10	0.39	0.97	0.13	−0.01	0.97	0.37	0.0035	0.0004	0.0039	0.0129
P6V2	0.29	0.78	1.07	−0.29	0.13	−0.09	−0.38	−0.13	0.0035	0.0091	0.0127	−0.0035
P6V3	0.29	−0.37	−0.08	0.01	0.13	−0.10	−0.08	−0.13	0.0035	−0.0019	0.0016	0.0012
P6V4	0.29	−0.18	0.11	0.65	0.13	0.13	0.77	0.23	0.0035	−0.0021	0.0014	0.0085
P6V5	0.29	−0.22	0.06	−0.60	0.13	0.09	−0.50	−0.60	0.0035	−0.0017	0.0019	−0.0059
P6V6	0.29	0.21	0.5	0.25	0.13	0.15	0.40	0.05	0.0035	0.0034	0.0069	0.0037
V1P7	0.12	0.17	0.29	0.74	0.09	0.01	0.75	0.35	0.0022	0.0012	0.0034	0.0098
V1P8	0.12	0.10	0.22	−0.34	0.09	−0.01	−0.34	−0.16	0.0022	0.0004	0.0026	−0.0023
V1V6	0.12	0.21	0.34	−0.48	0.09	0.15	−0.34	−0.34	0.0022	0.0034	0.0056	−0.0024
Mean	0.05	0.06	0.11	0.00	0.04	0.04	0.04	−0.04	0.0003	0.0006	0.0009	0.0010

**Table 7:** *Qi* values and genetic gains of different families.

Serial Number	Family	*Q_i_*	ΔG*_H_*-*TH* (%)	ΔG*_H_*-*DBH* (%)	ΔG*_H_*-*VI* (%)
1	P6P8	1.732	1.81	2.22	6.98
2	V1P7	1.706	1.32	−1.24	0.29
3	P5P8	1.670	4.15	9.84	24.57
4	P6V6	1.670	1.85	2.38	8.00
5	P6V4	1.660	1.83	2.40	8.31
6	P1V5	1.646	−5.02	−7.43	−19.81
7	P6V2	1.645	2.05	2.36	7.94
8	P2V6	1.639	2.57	6.20	17.31
9	P2P7	1.629	2.60	6.27	17.60
10	P4P8	1.619	−8.50	−10.90	−27.84
11	P1P8	1.606	−5.44	−7.57	−20.76
12	P6E1	1.604	1.95	2.49	9.28
13	P5V5	1.596	4.27	10.71	29.11
14	V1P8	1.578	1.42	−1.41	0.39
15	P4V4	1.543	−8.49	−11.92	−35.12
16	V1V6	1.540	1.47	−1.51	0.40
17	P4P7	1.539	−8.58	−11.80	−35.71
18	P6V3	1.529	2.04	2.74	10.89
19	P2P8	1.523	2.76	6.96	23.04
20	P1P7	1.457	−5.94	−8.70	−31.59
21	P3P8	1.442	−5.66	−6.77	−15.59
22	P6V5	1.438	2.17	3.03	13.99

**Table 8:** *Qi* values and genetic gains of different single plants.

Serial Number	Block	Family	*Q_i_*	ΔG_h_-*TH* (%)	ΔG_h_-*DBH* (%)	ΔG_h_-*VI* (%)
1	3	P5P8	1.704	4.48	4.73	6.69
2	2	P6P8	1.634	1.56	1.49	2.94
3	2	V1P8	1.633	1.09	−0.84	0.12
4	1	P2V6	1.612	1.94	4.25	6.83
5	1	P6P8	1.608	1.64	1.48	3.06
6	3	V1P7	1.601	1.15	−0.84	0.13
7	2	P6V6	1.594	1.44	1.71	3.57
8	2	P5V5	1.593	3.60	6.42	9.90
9	2	V1V6	1.577	1.16	−0.86	0.13
10	1	P6V4	1.574	1.53	1.66	3.59
11	1	P2P7	1.571	2.18	4.06	7.03

**Table 9:** The parental combinations of 22 full-sibling families.

Family	Female Parent	Male Parent	Family	Female Parent	Male Parent
P1P7	*F. pennsylvanica ‘Hong 1’*	*F. pennsylvanica ‘Jinguan’*	P5V5	*F. pennsylvanica ‘Hong 5’*	*F. velutina ‘J30’*
P1P8	*F. pennsylvanica ‘Hong 1’*	*F. pennsylvanica ‘Lula 5’*	P6E1	*F. pennsylvanica ‘Lula 6’*	*F. excelsior ‘Jinzhi’*
P1V5	*F. pennsylvanica ‘Hong 1’*	*F. velutina ‘J30’*	P6P8	*F. pennsylvanica ‘Lula 6’*	*F. pennsylvanica ‘Lula 5’*
P2P7	*F. pennsylvanica ‘Hong 2’*	*F. pennsylvanica ‘Jinguan’*	P6V2	*F. pennsylvanica ‘Lula 6’*	*F. velutina ‘J16’*
P2P8	*F. pennsylvanica ‘Hong 2’*	*F. pennsylvanica ‘Lula 5’*	P6V3	*F. pennsylvanica ‘Lula 6’*	*F. velutina ‘J19’*
P2V6	*F. pennsylvanica ‘Hong 2’*	*F. velutina ‘J31’*	P6V4	*F. pennsylvanica ‘Lula 6’*	*F. velutina ‘J29’*
P3P8	*F. pennsylvanica ‘Hong 3’*	*F. pennsylvanica ‘Lula 5’*	P6V5	*F. pennsylvanica ‘Lula 6’*	*F. velutina ‘J30’*
P4P7	*F. pennsylvanica ‘Hong 4’*	*F. pennsylvanica ‘Jinguan’*	P6V6	*F. pennsylvanica ‘Lula 6’*	*F. velutina ‘J31’*
P4P8	*F. pennsylvanica ‘Hong 4’*	*F. pennsylvanica ‘Lula 5’*	V1P7	*F. velutina ‘Qingbi’*	*F. pennsylvanica ‘Jinguan’*
P4V4	*F. pennsylvanica ‘Hong 4’*	*F. velutina ‘J29’*	V1P8	*F. velutina ‘Qingbi’*	*F. pennsylvanica ‘Lula 5’*
P5P8	*F. pennsylvanica ‘Hong 5’*	*F. pennsylvanica ‘Lula 5’*	V1V6	*F. velutina ‘Qingbi’*	*F. velutina ‘J31’*

**Table 10:** Survival rate of families.

Family	Number of Trees	Survival Rate (%)	Family	Number of Trees	Survival Rate (%)
P1P7	57	95.00	P5V5	57	95.00
P1P8	59	98.33	P6E1	59	98.33
P1V5	58	96.67	P6P8	58	96.67
P2P7	59	98.33	P6V2	59	98.33
P2P8	56	93.33	P6V3	56	93.33
P2V6	58	96.67	P6V4	58	96.67
P3P8	55	91.67	P6V5	55	91.67
P4P7	60	100.00	P6V6	60	100.00
P4P8	56	93.33	V1P7	56	93.33
P4V4	60	100.00	V1P8	60	100.00
P5P8	54	90.00	V1V6	54	90.00

**Table 11:** Phenotypic/genetic parameters and model calculation formulas. *VI*: volume index; *TH*: tree height; *DBH*: diameter at breast height; *X_ijK_*: performance of the *k*-th tree in family *i* within block *j*; *μ*: general mean; *F_i_*: effect of the *i*-th family; *B_j_*: effect of the *j*-th block; *FB_ij_*: interaction effect between family *i* and block *j*; *e_ijk_*: random error; *PCV*: phenotypic coefficient of variation; *σ_p_*^2^: phenotypic variance component; *GCV*: genotypic coefficient of variation; *σ_g_*^2^: genetic variance component; *X*: mean of trait; *H*^2^: family heritability; *h*^2^: individual tree heritability; *σ_F_*^2^: family variance component; *σ_FB_*^2^: family × block interaction variance component; *σ_e_*^2^: error variance component; *B*: number of blocks; *N*: total number of families. ΔG*_H_*: genetic gain from family selection; ΔG*_h_*: genetic gain from individual tree selection; *S*: selection differential; *H*: heterosis; $\bar{X}$ *_Fi_*: mean value of family I; $\bar{OP}$: mean value of all families sharing the same parents as family *i*; *GCA_P1i_*: general combining ability of the *i*-th female parent; $\bar{X}$*_P1_*: mean trait value of all families with female parent *i*; *GCA_P2j_*: general combining ability of the *j*-th male parent; $\bar{X}$*_P2j_*: mean trait value of all families with male parent *j*; $\bar{X}$: mean trait value of all families; *GCA_ij_*: general combining ability of the fixed family of female parent *i* and male parent *j*; *SCA_ij_*: specific combining ability of the hybrid combination of female parent *i* and male parent *j*; $\bar{X}$*_ij_*: mean trait value of the hybrid combination of female parent *i* and male parent *j*; *Q_i_*: comprehensive evaluation index value; *x_ij_*: mean value of trait *j* for family *i*; *x_jmax_*: maximum value of trait *j*; *n*: number of traits.

Number	Parameter/Model	Formula
1	Volume index [79]	*VI* = *TH* × *DBH*^2^
2	Linear model [80]	*X_ijk_* = *μ* + *F_i_* + *B_j_* + *FB_ij_* + *e_ijk_*
3	Coefficient of variation [81]	$PCV=\frac{\sqrt{\sigma_{p}^{2}}}{X}\times 100$
4		$GCV=\frac{\sqrt{\sigma_{g}^{2}}}{X}\times 100$
5	Heritability [82]	*H*^2^ = *σ_F_*^2^/(*σ_F_*^2^ + *σ_FB_*^2^/*B* + *σ_e_*^2^/*NB*)
6		*h*^2^ = 4*σ_F_*^2^/(*σ_F_*^2^ + *σ_FB_*^2^ + *σ_e_*^2^)
7	Genetic gain [42]	ΔG*_H_* = *H*^2^*S*/*X*
8		ΔG*_h_* = *h*^2^*S*/*X*
9	Heterosis [82]	*H* = ($\bar{X}$*_Fi_* − $\bar{OP}$)/$\bar{OP}$ × 100%
10	Combining ability [83]	*GCA_P1i_* = $\bar{X}$*_P1i_* − $\bar{X}$
11		*GCA_P2j_* = $\bar{X}$*_P2j_* − $\bar{X}$
12		*GCAij* = *GCA_P_*_1i_ + *GCA_P2j_*
13		*SCA_ij_* = $\bar{X}$*_ij_* − $\bar{X}$ − *GCA_i_* − *GCA_j_*
14	Comprehensive evaluation [83]	$Q_{i=}\sqrt{\sum_{i=1}^{n}a_{i}}$; $a_{i=}\frac{x_{ij}}{x_{jmax}}$

## Data Availability

The data underlying this article are available in the article and in its Supplementary Materials.

## Supplementary Materials

The following supporting information can be downloaded at: https://www.mdpi.com/article/10.3390/plants14162601/s1, Table S1: 14 pairs of primer information; Figure S1: the electrophoresis for some screened SSR primers.
