# Supplementary material for: Predicting Heterosis and Selecting Superior Families and Individuals in Fraxinus spp. Based on Growth Traits and Genetic Distance Coupling
Source: Plants (Basel). 2025 Aug 21;14(16):2601. doi: 10.3390/plants14162601 (PMC12388951; doi:10.3390/plants14162601)
Supplement: Supplementary file 1 [file plants-14-02601-s001.zip › plants-3793514-supplementary.pdf]

Supplementary Materials:

Table S1 14 pairs of primer information

Primer information	Forward primer(5′-3′)	Rever primer(5′-3′)	Fragment size/bp	Base pairs
SSR 82	TTGACTCGTGTTTAGGGATGAAT	AGCTCTTGAAGGGAAAATTTGAA	154	CAG(3*5)
SSR 93	GAAAAGGAGGAGAGTGGGAATAC	GCTCCATTTCACTTCAACTCTTC	142	GAA(3*5)
SSR 95	AGAATAGATGAGGATGAAGGGGA	CTAACTCATCCCTCTGCGAAAC	150	GAA(3*6)
SSR 112	CCATTGTCAATTTGCAGATTCTT	GTCTGGAAATGTTGATCCTGAAA	141	ATT(3*5)
SSR 120	GAATGATCTGGTTGCTGAATACC	AGAGATTTGGACATCTGATGGAA	136	GAG(3*6)
SSR 144	GATAGTGGGGGAAGAATAAGTCC	TCATTCCAACTCAATGAACTCCT	104	AGC(3*5)
SSR 147	GGATAGTGGGGGAAGAATAAGTC	CTCATTCCAACTCAATGAACTCC	106	AGC(3*5)
SSR 167	TGAGCAAATGTGAAGACCGTAG	TAATTTCATCCACCAGTTTCCAC	156	TGG(3*5)
SSR 186	TCTTCACGTCTTCTGTTTGTTCA	GAAAACGTGTGAATGAGTTTGGT	114	GTAGAA(6*4)
SSR 187	TCGATCTTTCCATCTAAACAAGC	AACGTGTGAATGAGTTTGGTTTT	151	GTAGAA(6*4)
SSR 202	AGTTTTCACCGCTTTCAGTGTTA	GGGAATGAACATGAGTTTCAGTA	113	GTG(3*5)
SSR 203	GTTATCAGTAGATGCAACCGCAC	AACACCGGTTTTCAACATTTCT	154	GTG(3*5)
SSR 208	CCTCCTATTGAATCATTCGCTTA	ATTTTGATTTCCCTCCTCTGAAG	141	CAA(3*5)
SSR 213	GACAACATGCCTAAATTGGACTC	AATTCTGAACTTCAAGGTGGGAT	92	CGG(3*5)

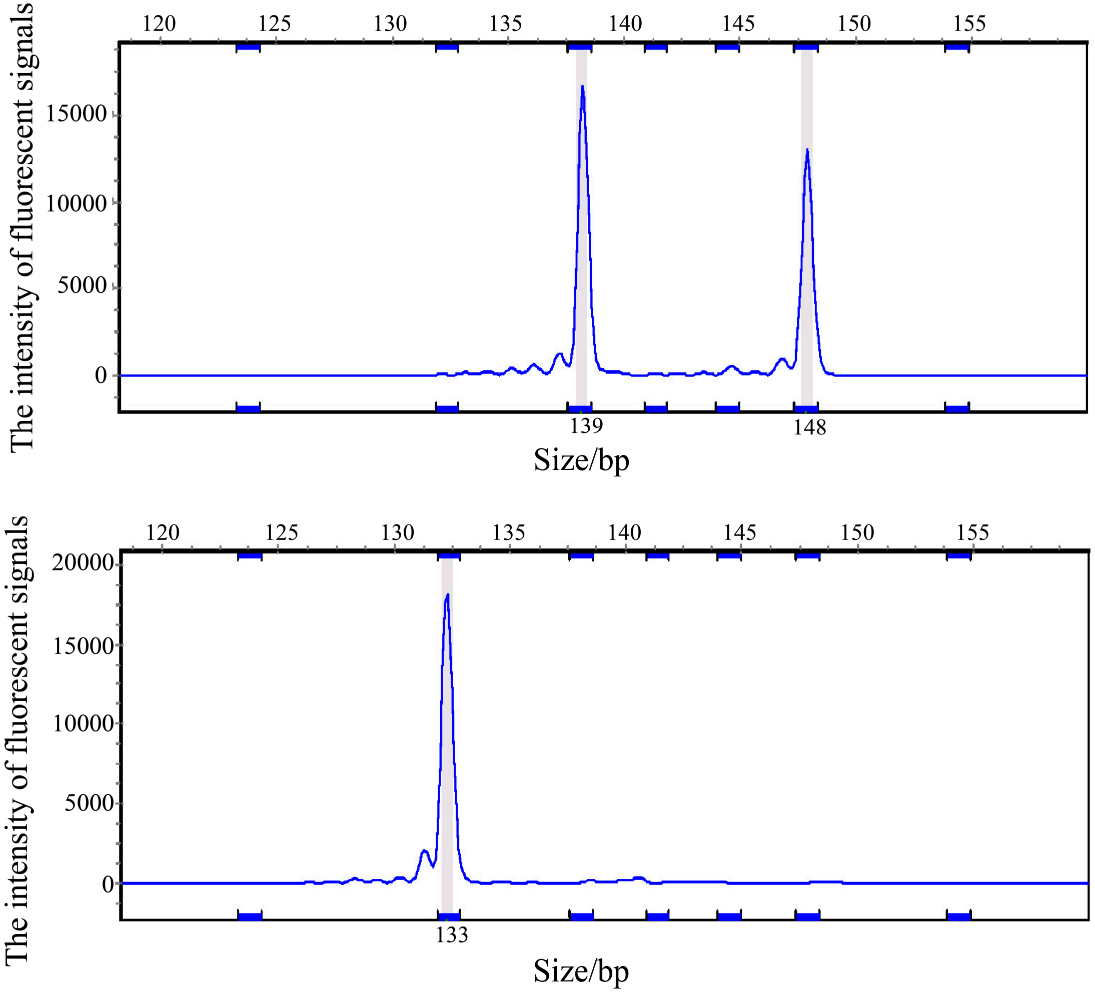

Figure S1 The electrophoresis for some screened SSR primers
